# Pluripotent stem cell‐derived organoids: A brief history of curiosity‐led discoveries

**DOI:** 10.1002/bies.202400105

**Published:** 2024-08-05

**Authors:** Madeline A. Lancaster

**Affiliations:** ^1^ MRC Laboratory of Molecular Biology Cambridge UK

**Keywords:** development, morphogenesis, organoids, stem cells, tissue engineering

## Abstract

Organoids are quickly becoming an accepted model for understanding human biology and disease. Pluripotent stem cells (PSC) provide a starting point for many organs and enable modeling of the embryonic development and maturation of such organs. The foundation of PSC‐derived organoids can be found in elegant developmental studies demonstrating the remarkable ability of immature cells to undergo histogenesis even when taken out of the embryo context. PSC‐organoids are an evolution of earlier methods such as embryoid bodies, taken to a new level with finer control and in some cases going beyond tissue histogenesis to organ‐like morphogenesis. But many of the discoveries that led to organoids were not necessarily planned, but rather the result of inquisitive minds with freedom to explore. Protecting such curiosity‐led research through flexible funding will be important going forward if we are to see further ground‐breaking discoveries.

## Graphical abstract

Pluripotent stem cell‐derived organoids are enabling new insights into human biology and disease. This exciting field did not necessarily start with translational potential in mind. Rather it was curiosity into self‐organization and the evolution of embryoid bodies with increasingly complex organ‐like morphogenesis that enabled the advanced PSC‐organoids of today.

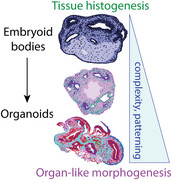

## INTRODUCTION

There are many fundamental biological differences between humans and other organisms. Nothing exemplifies this better than clinical trial failure rates. At least 90% of drug candidates fail in clinical trials despite successes in animal disease models.^[^ ^1^ ^]^ For example, rodent models of spinal cord injury have demonstrated functional recovery in at least 69 studies of various therapeutics,^[^ ^2^ ^]^ yet human trials have so far been rather disappointing, with one such strategy going as far as phase III but failing due to lack of therapeutic effect.^[^ ^3^ ^]^ Reasons for this failure are myriad, but can essentially be boiled down to human‐specific differences, be they differences in drug kinetics, toxicity, efficacy, or even more fundamental differences in human biology.

This highlights the need for human models. Naturally, these must be in vitro models, made up of human cells or tissues of varying complexities. Organoids represent the more complex of these, being made up of multiple cell types and exhibiting a tissue architecture and function seen in the actual organ.^[^ ^4^ ^]^ The early organoids of Sato et al.,^[^ ^5^ ^]^ and Ootani et al.^[^ ^6^ ^]^ in 2009, were derived from already mature tissue of the gut by isolating and culturing adult stem cells in a three‐dimensional basement membrane protein‐rich gel. These intestinal organoids demonstrated the ability of even committed adult progenitors to form functional tissue in a dish. However, unlike the rapid turnover of the intestine, most other organs do not contain such potent progenitors. Hence, to model such tissues, embryonic or induced PSCs can be differentiated to the relevant cell types in vitro. This makes PSC‐derived organoids fundamentally different from adult‐derived organoids. The use of PSCs introduces variability in the types of cells that are made, but it also enables impressive self‐organization and can capture the complex processes of patterning and morphogenesis.

PSC‐derived organoids are possible because of the remarkable robustness of tissue development, even in the absence of the embryonic environment. But such self‐organization was not necessarily predicted even by those who first established these models. Instead, it was through curiosity‐led studies simply aimed at observing developing cells and tissues that such organoids were made possible. And it was rather difficult to predict that these in vitro oddities would turn out to be such a fundamental tool for human biology and drug discovery. Like so many ground‐breaking discoveries, PSC‐derived organoids were borne out of curiosity and a search for fundamental insight, without constraints or even a clear plan for translational impact. In this es, I provide a historical perspective highlighting key advances from early tissue reaggregation studies to embryoid bodies (EBs) and in vitro histogenesis, and finally to more advanced organ‐like morphogenesis. I argue that freedom to explore was an integral part of enabling this technology, and that going forward, scientific curiosity should be protected rather than stifled by a push for immediate translation.

## EARLY FOUNDATIONS OF THE FIELD

Although the term “organoid” as it is used today is a relatively recent one, self‐organizing in vitro tissues were described as early as the beginning of the last century. Wilson demonstrated that individual cells and small masses of cells could be physically isolated from a particular species of sea sponge, and upon reaggregation would form a new sponge.^[^ ^7^ ^]^ Then, in the 1940s, Barth and Holtfreter discovered that the amphibian animal cap could differentiate into neural tissue even when isolated from its embryonic environment.^[^ ^8^, ^9^, ^10^ ^]^ For many years, Holtfreter and others searched fervently for the inducer, suggesting even rather bizarre possibilities such as sand.^[^ ^10^ ^]^ A hint that there may be an intrinsic ability of cells to form such tissues came from the work of Moscona in 1952 while at the Strangeways Research Laboratory in Cambridge who performed dissociation‐reaggregation of embryonic chick tissues,^[^ ^11^, ^12^ ^]^ revealing a remarkable ability of completely isolated cells to re‐form new tissues. Although these experiments were performed in the middle of the last century, the results led to debates similar to those currently at the forefront of developmental biology. As Weiss and Taylor wrote in 1960^[^ ^13^ ^]^:

“The results re‐emphasize internal ‘self‐organization’ as one of the most basic problems in the study of development, in contra‐distinction to contemporary preoccupation with external ‘inductions.’”

Such a statement is as true now as it was then. Much of current developmental biology has focused on extrinsic signaling cues as the key determinants of cellular behaviors, yet these self‐organizing cellular aggregates demonstrate a robust, intrinsic developmental programme whose mechanism is still largely unclear.

Dissociation‐reaggregation studies provided important insight into how adhesion proteins like cadherins allow cells to sort out into different domains.^[^ ^14^ ^]^ But the intrinsic developmental programmes that govern such self‐organization remained unknown, and for the past several decades, dissociation‐reaggregation studies were largely ignored. The connection to stem cell biology and its translational potential was certainly not evident at the time.

## EMBRYOID BODY BEGINNINGS

As cultures of embryonic stem cells (ESCs) became possible,^[^ ^15^, ^16^, ^17^ ^]^ attention turned to ways of differentiating stem cells to particular cell types with therapeutic relevance, again with a predominant focus on external signaling and inductive cues.

As more insights into morphogenetic cues became clear from embryonic studies of organizers and the role of patterning factors, it became possible to direct the differentiation of PSCs to various organ identities, such as cardiomyocytes,^[^ ^18^ ^]^ pancreatic cells,^[^ ^19^ ^]^ and neurons.^[^ ^20^, ^21^, ^22^ ^]^ The fact that PSCs could be maintained in culture and in theory differentiated to any cell type of the body held great promise for regenerative medicine. There was a lot of hope but unfortunately also hype, and many have argued that the stem cell field has failed to live up to its promises.^[^ ^23^ ^]^ This may reflect the fact that extrinsic inductive signals are simply not sufficient to generate bona fide functional cells. In fact, we now know that cells have a more faithful identity when they develop within a tissue context.^[^ ^24^ ^]^

**FIGURE 1:**
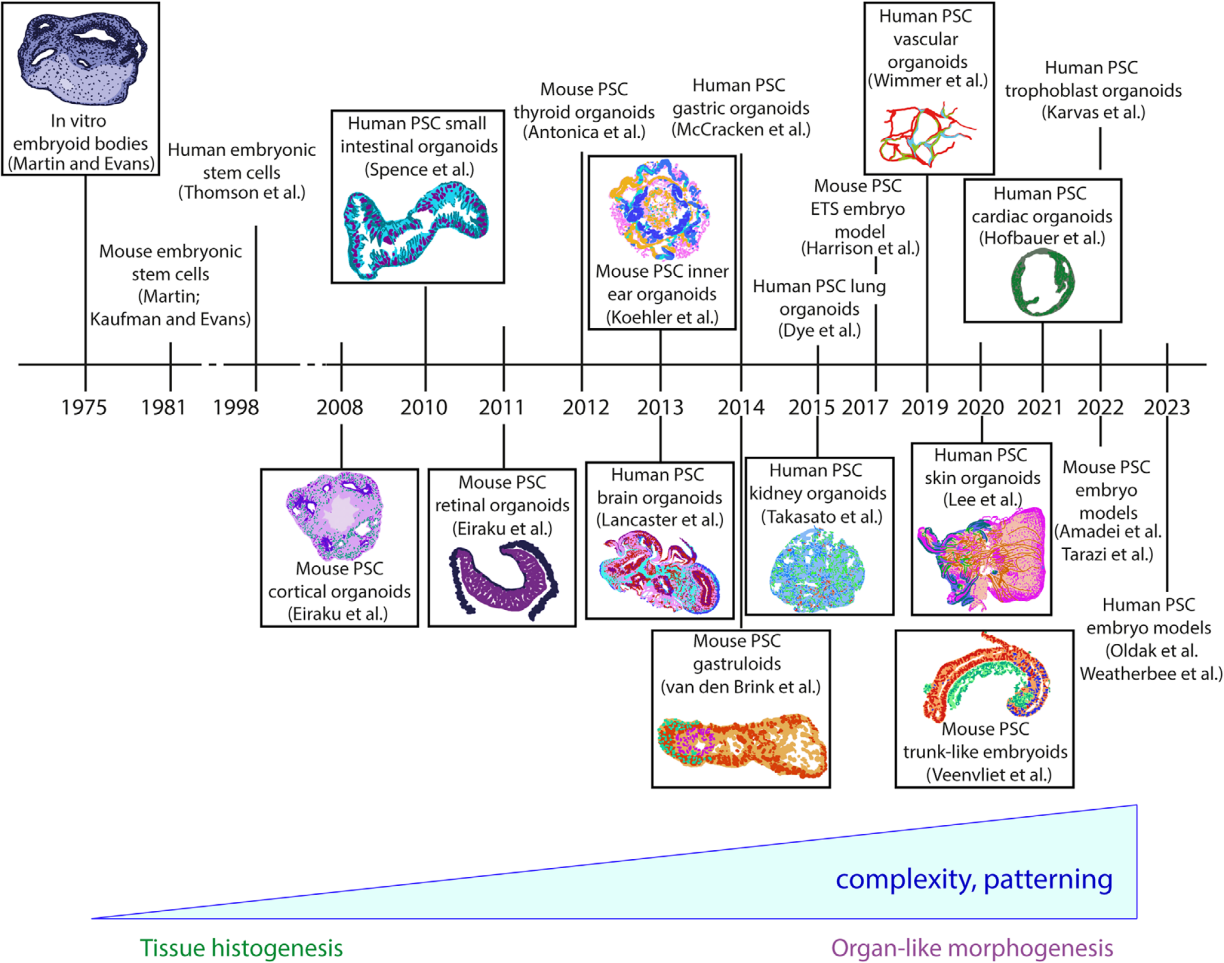
A brief history of pluripotent stem cell (PSC)‐derived organoids. A timeline of PSC‐derived tissues and their evolution from embryoid bodies. A selection of methods is illustrated with drawings based on actual data shown in the referenced publications. The progression from histogenesis to organ‐like morphogenesis in more complex organoids with regional patterning (i.e., brain/cerebral organoids, gastruloids, kidney organoids, skin organoids, and trunk‐like embryoids) is highlighted below.

Early work on 3D differentiation involved structures called EBs^[^ ^25^, ^26^ ^]^ (Figure 1), often by aggregating single cell suspensions of PSCs in a hanging drop of media. EBs exhibit spontaneous differentiation to give rise to rudimentary structures of endo‐, meso‐, and ectoderm, representing the three germ layers of the embryo.^[^ ^27^ ^]^ While early studies recognized the histogenic potential of EBs to form structures reminiscent of tissues,^[^ ^25^ ^]^ their heterogeneity and complexity made it difficult to control cell differentiation. For example, while contractile cardiomyocytes could be observed to form spontaneously in EBs^[^ ^28^ ^]^ their appearance was rather unpredictable.^[^ ^29^ ^]^

## DIRECTING 3D HISTOGENESIS

A focus on more control over the cell types generated in EBs led many researchers to perform such 3D differentiations only transiently and to plate EBs on 2D substrates where the cells could be more evenly exposed to inductive cues to force differentiation down particular lineages.^[^ ^30^ ^]^ Such was the approach of several groups, including the lab of Yoshiki Sasai, whose work laid important foundations for the organoid field. In the early 2000s, Sasai and others were largely focused on determining inductive cues for directed differentiation. For example, EBs grown in a serum‐free medium with Wnt and Nodal antagonists and then exposed to adherent culture generated forebrain neurons.^[^ ^31^ ^]^ Likewise, application of other inductive cues after initial directed neural differentiation generated cells of the retina^[^ ^32^ ^]^ and cerebellum.^[^ ^33^ ^]^ However, such strong extrinsic patterning and the 3D‐to‐2D approach naturally disrupts self‐organization processes and the resultant cell clumps did not display signs of histogenesis.

In 2008, Sasai's group demonstrated that rather than plating 2D substrates, neural induced mouse EBs maintained in 3D culture could exhibit histogenic properties reminiscent of the embryonic cortex^[^ ^34^ ^]^ (Figure 1). The resultant structures exhibited a progenitor zone and a neural plate, similar to but larger than 2D neural rosettes, and reminiscent of previously described structures seen in neuroectodermal regions of teratomas and EBs^[^ ^26^ ^]^, highlighting the fact that the foundations of PSC‐derived organoids were already evident in these earlier structures. While more advanced patterning and organ suprastructure was still lacking, it highlighted the robust nature of neural ectoderm to form a polarized neuroepithelium in 3D.

Turning to the endoderm, in 2010 Jason Spence and James Wells produced PSC‐derived gut organoids by inducing human PSCs in 2D culture to definitive endoderm and then directing them to a hindgut fate.^[^ ^35^ ^]^ Cells self‐organized into 3D growths that budded from the dish. Rather than aspirate these spheroids with media changes as would have typically been done previously, the authors maintained them in 3D culture conditions similar to those used for adult‐derived gut organoids. The result was the formation of a convoluted epithelium reminiscent of embryonic intestinal epithelium (Figure 1), but unlike adult‐derived organoids the PSC‐derived hindgut tissues also developed associated mesenchyme.

Sensory organs were also an early tissue type of focus. In 2011, the Sasai lab developed impressive retinal structures from mouse EBs.^[^ ^36^ ^]^ As with cortical directed EBs, the authors observed that maintenance of retinal induced mouse EBs in 3D culture resulted in more advanced histogenesis, but in this case the tissue architecture went beyond that seen in the more rudimentary EBs or teratomas. The retinal tissues displayed impressive stratified cytoarchitecture with an outer nuclear layer, inner nuclear layer, and ganglion cell layer composed of the major retinal cell types exhibiting advanced morphologies. Turning to another type of sensory epithelium, Koehler et al. in 2013 directed mouse EBs to inner ear fate which developed impressive tissue cytoarchitecture when similarly maintained in 3D self‐organizing culture rather than simple 2D culture.^[^ ^37^ ^]^ This work highlighted the power of self‐organization, and in 2013 Sasai introduced the concept of “cytosystems dynamics” indicating a shift in focus more in favor of the viewpoint of Weiss and Taylor, suggesting a more holistic approach may be needed to uncover the core modules governing self‐organization^[^ ^38^ ^]^:

“Multicellular systems involve huge numbers of regulatory components, and the complexity of these in multidimensional networks is beyond comprehension. Such highly robust and regulative phenotypes could never be explained by strict control of each regulatory component in the system.”

Self‐organization is an emergent property that cannot be understood or recapitulated by simply combining the parts. This makes it in many ways a black box, but nonetheless a powerful tool to grow, rather than build, potentially any tissue of the body.

As with many areas of regenerative medicine, experiments in mouse or other model organisms provided the foundation on which to develop human systems. Human EBs similarly subjected to directed differentiation to retinal identity also exhibited striking histogenesis with stratified retinal cell types.^[^ ^39^ ^]^ In the same study that described cortical induced mouse EBs kept in 3D, human ESCs were similarly subjected to the same inductive cues and also formed intriguing cortical zones, though the authors reverted to 2D plating of the human EBs rather than maintaining them in 3D culture.^[^ ^34^ ^]^ These studies demonstrate a gradual move away from the rather heavy‐handed extrinsic control over differentiation that was the norm at the time. Although Yoshiki Sasai's work still focused on inductive cues to direct differentiation, the tissues his lab produced demonstrated the power of the 3D environment, a realization that began to permeate the field.

## MORPHOGENESIS IN VITRO

In vivo studies have demonstrated that neural differentiation is the default for PSCs,^[^ ^40^ ^]^ and in vitro cultures have revealed that even serum can influence differentiation and direct nonneural identities.^[^ ^22^, ^41^ ^]^ Thus, while the early PSC‐organoids utilized small molecules and growth factors to direct differentiation, evidence was building that directed differentiation may not be necessary in the case of neural identity. Because such extrinsic manipulation could override intrinsic developmental programs, an undirected 3D differentiation aimed at supporting, rather than directing, differentiation could provide more extensive self‐organization.

**FIGURE 2:**
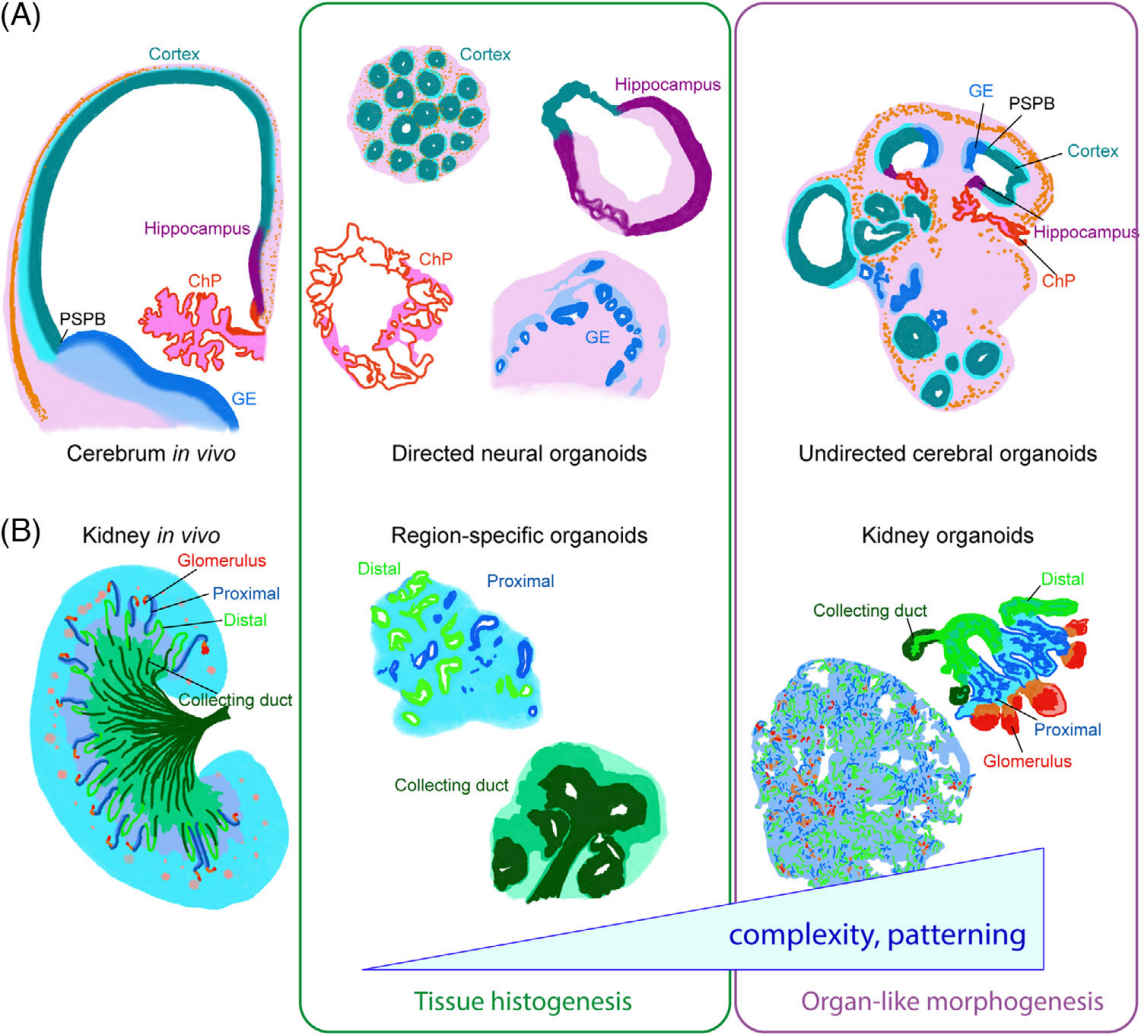
Various organoids model different degrees of organ structure and patterning. (A) A diagram of a coronal section of one hemisphere of the developing human cerebrum showing the organization of dorsal and ventral regions progressing from the choroid plexus (ChP) to the hippocampus and cortex, to the pallial‐subpallial boarder (PSPB) and the ventral ganglionic eminences (GE). These individual regions can be modeled in guided organoids made through directed differentiation using signaling factors, or can develop intrinsically in close proximity in a similar pattern to in vivo in undirected brain/cerebral organoids. Images are drawings based on actual data shown in: Altman and Bayer^[^ ^94^ ^]^ (in vivo), Paşca et al.^[^ ^95^ ^]^ (cortical organoids), Pellegrini et al.^[^ ^96^ ^]^ (ChP organoids), Sakaguchi et al.^[^ ^97^ ^]^ (hippocampal organoids), Bagley et al.^[^ ^98^ ^]^ (ventral forebrain organoids), and Renner et al.^[^ ^44^ ^]^ (undirected cerebral organoids). (B) A diagram of a kidney showing the hierarchical structure of the nephron and collecting ducts. Specific regions can be modeled independently in single region organoids, but can also be modeled together where they form regionalized tubules like those found in the developing kidney in vivo. Images are drawings based on actual data shown in: Taguchi et al.^[^ ^55^ ^]^ (metanephric mesenchyme organoids), Xia et al.^[^ ^54^ ^]^ (ureteric bud organoids), and Takasato et al.^[^ ^57^ ^]^ (kidney organoids).

Originally named after the Latin for brain, so‐called cerebral organoids generated with minimal guidance were shown to exhibit not just isolated neural tissues, but a variety of brain regional identities within the same mass, including retinal, forebrain, choroid plexus, and mid/hindbrain tissues.^[^ ^42^ ^]^ Although current nomenclature consensus^[^ ^43^ ^]^ is such that “cerebral” should denote the neuroanatomic meaning referring to the telencephalon rather than the etymological origin of the word, the original cerebral organoids exhibited not only isolated histogenesis of cerebral structures but also a degree of organ patterning (Figure 2). For example, forebrain structures exhibited rostral‐caudal (i.e., frontal versus occipital lobes) and dorsal‐ventral patterning with ventrally produced interneurons migrating into the adjacent dorsal region,^[^ ^42^ ^]^ just as in vivo. Likewise, mediolateral organization could be observed with the cerebrospinal fluid‐producing choroid plexus transitioning into hem neuroepithelium and stratified cortical tissue with fluid‐filled ventricles,^[^ ^44^ ^]^ just as in vivo. This spatial organization demonstrated an intrinsic axial patterning going beyond local cell–cell interactions seen in histogenesis and suggesting even incredibly complex morphogenetic patterning could be accomplished in vitro.

The potential impact of PSC‐derived organoids in the biomedical sphere was not necessarily clear at first. Cerebral organoids provided a demonstration of that potential when they were used to model a human condition, microcephaly. By starting with patient‐derived induced pluripotent cells (iPSCs), the organoids revealed a pathogenic mechanism in which neural stem cells became depleted resulting in fewer neurons produced.^[^ ^42^ ^]^ This condition is not well modeled in mice, so it provided a proof‐of‐concept that brain organoids could uncover human‐specific biology. Perhaps more importantly, it established iPSC‐derived organoids as a tool for understanding human disease, and opened the flood‐gates to a new era of disease modeling and drug discovery.^[^ ^45^, ^46^ ^]^

Since the early reports of PSC‐derived 3D tissues and organoids, numerous organs including thyroid,^[^ ^47^ ^]^ stomach,^[^ ^48^ ^]^ lung,^[^ ^49^ ^]^ skin,^[^ ^50^ ^]^ heart,^[^ ^51^ ^]^ placenta,^[^ ^52^ ^]^ and vasculature,^[^ ^53^ ^]^ have been modeled in a similar fashion (Figure 1), often through gently guiding EBs or dense 2D cultures to the organ precursor of interest and allowing self‐organization to take over. For example, to generate organoids of the kidney, several labs devised ways to first direct differentiation to the intermediate mesoderm and then to ureteric bud,^[^ ^54^ ^]^ metanephric mesenchyme,^[^ ^55^ ^]^ or both.^[^ ^56^ ^]^ By first establishing the primordial tissue and providing a permissive culture environment, these tissues could self‐organize and form impressive regionalized nephric tubules and glomeruli^[^ ^57^ ^]^ (Figure 2). More recently, these principles have been taken to a new level with the development of embryo‐like models, including gastruloids,^[^ ^58^, ^59^ ^]^ embryonic trunk‐like tissues with somites,^[^ ^60^ ^]^ and models of the peri‐ and post‐implantation embryo.^[^ ^61^, ^62^, ^63^, ^64^, ^65^, ^66^ ^]^ Here too, the concept of gentle guidance with a strong reliance on self‐organization is leading to impressive new models with axial patterning reflecting true morphogenetic processes in vitro.

## THE IMPORTANCE OF CURIOSITY AND FREEDOM

While it is difficult to know what on a personal level led many researchers in this field to begin experimenting with more and more complex in vitro tissues, it is rather safe to say that all had one thing in common: curiosity. Yoshiki Sasai's publication record indicates a highly inquisitive mind and a thirst for fundamental understanding of developmental biology.^[^ ^67^ ^]^ Similarly, when I began my postdoc in 2010 in the laboratory of Juergen Knoblich, at the Institute for Molecular Biotechnology in Austria (IMBA), we were driven by a desire for fundamental insight, largely unfettered by concerns about work packages or promises to cure diseases. Knoblich and I sketched out a rather rough outline to develop an in vitro assay to test various orthologs of drosophila genes of interest in mouse neural stem cells.

Like many discoveries in science, an aspect of serendipity played a part, and it is likely that similar serendipity was at play for others developing PSC‐derived organoids.^[^ ^68^ ^]^ But it was only because of academic freedom that we were able to follow where luck would have it. Because I was the first in the lab to culture stem cells, I did not have certain necessary reagents on hand, so I scrounged around for tissue culture plates and coating reagents. These were suboptimal, likely expired and inactive. Lacking experience with stem cell culture, I went ahead anyway, and my first attempts, which involved culturing mouse neuroepithelial cells that I had dissected from E9.5 embryos, failed to form attached neural rosettes. Instead, 3D structures appeared with evident lumens, reminiscent of the neural tube.^[^ ^69^ ^]^ I was completely enthralled that such complex structures could form in the absence of any extrinsic guidance, setting off the curiosity‐driven investigation that led to cerebral organoids.

## FUTURE PROSPECTS

With organoids becoming an important part of disease modeling and drug development portfolios, their translational utility is now evident. Patient‐derived iPSCs are now routinely used to derive organoids for a range of human conditions, including Hirschsprung's disease,^[^ ^70^ ^]^ polycystic kidney disease,^[^ ^71^ ^]^ autism,^[^ ^72^ ^]^ epilepsy,^[^ ^73^ ^]^ and tuberous sclerosis,^[^ ^74^ ^]^ to name a few. Taking this to a new level, Timothy syndrome, a debilitating disorder presenting with autism and epilepsy, has not only been modeled in neural organoids^[^ ^75^ ^]^ but a new antisense oligonucleotide therapeutic strategy has been developed that has enormous potential for patients.^[^ ^76^ ^]^ Thus, organoids are providing not only new understanding, but even new therapeutic avenues.

The power of certain organoids to undergo tissue histogenesis and even organ‐like morphogenesis all through self‐organizing principles is impressive. However, with less extrinsic control comes more intrinsic variability. Thus, while a greater reliance on self‐organization can lead to impressive morphogenesis, outcomes can be variable. This fact has influenced the field to begin swinging again in the other direction, with more recent methods generating more simplified structures and exerting greater control through more inductive signals aimed at achieving greater homogeneity.^[^ ^77^, ^78^, ^79^ ^]^ History seems to be repeating itself with such heavily directed approaches resulting in a reduction of structural complexity, and a loss of morphogenesis.^[^ ^80^ ^]^ At the same time, new approaches combining directed differentiation with fusion of organoids of different identities in so‐called assembloids^[^ ^70^, ^81^, ^82^ ^]^ are adding new layers of complexity. It is important to remember that in many cases organs do not develop through fusion of different tissues, but rather through co‐development of tissues in proximity to set up the proper organization of different regions within and across organs (Figure 2). Such intrinsic patterning has already been seen in multi‐region brain organoids,^[^ ^44^ ^]^ neuromuscular junction organoids,^[^ ^83^ ^]^ skin organoids,^[^ ^50^ ^]^ and kidney organoids,^[^ ^57^ ^]^ for example. Going forward, it will be important to find ways to accomplish this morphogenesis in a predictable fashion that is reproducible from organoid to organoid.

The combination of organoids with other technologies such as bioengineering and gene editing is further expanding the potential of these tissue models. CRISPR provides an efficient method for precise genetic engineering and can yield functional insight into genes of interest^[^ ^84^, ^85^ ^]^ and uncover pathogenic mechanisms in disease modeling.^[^ ^86^ ^]^ Fluid‐flow in the form of bioreactors^[^ ^42^ ^]^ or micro/millifluidics^[^ ^87^ ^]^ can provide environments conducive to tissue growth and maturation, vascularization, and patterning.^[^ ^88^ ^]^ Likewise, the combination of organoids with organ‐on‐chip technologies^[^ ^89^, ^90^ ^]^ can provide environmental control and cross‐talk between different organoids. Furthermore, various materials can impose different conformations on organoids,^[^ ^91^, ^92^, ^93^ ^]^ which is enough to change their differentiation and maturation. These types of bioengineering approaches can take organoids in new directions and will no‐doubt be vital in going from organoid to in vitro organ, but as with all extrinsic manipulations, the balance between engineering and self‐organization will be important to bear in mind.

Thus, the debate between self‐organization and induction seems to be alive and well, and the pendulum will likely continue to swing between the two in the future. The important thing will be to maintain a sphere of academic freedom, so that curious minds can continue to tinker without entirely knowing where it will take them. To enable that, it is vital that core funding and grant schemes are provided to talented scientists without necessarily telling them what to do. The recent trend of granting agencies demanding immediate translational impact will do just the opposite. True impact cannot be predicted or directed, but comes from providing a nurturing, permissive environment to enable the very same self‐organizing principles at the heart of organoids themselves.

## AUTHOR CONTRIBUTIONS

Madeline A. Lancaster conceived and wrote the manuscript.

## CONFLICT OF INTEREST STATEMENT

Madeline A. Lancaster is an inventor on patents related to brain organoids and is cofounder of a:head bio.

## Data Availability

Data sharing is not applicable to this article as no new data were created or analyzed in this study.

## References

[1] Sun, D. , Gao, W. , Hu, H. , & Zhou, S. (2022). Why 90% of clinical drug development fails and how to improve it? Acta Pharmaceutica Sinica B, 12, 3049–3062. 10.1016/j.apsb.2022.02.002 35865092 PMC9293739

[2] Ulndreaj, A. , Badner, A. , & Fehlings, M. G. (2017). Promising neuroprotective strategies for traumatic spinal cord injury with a focus on the differential effects among anatomical levels of injury. F1000Research, 6, 1907. 10.12688/f1000research.11633.1 29152227 PMC5664995

[3] Koda, M. , Hanaoka, H. , Fujii, Y. , Hanawa, M. , Kawasaki, Y. , Ozawa, Y. , Fujiwara, T. , Furuya, T. , Ijima, Y. , Saito, J. , Kitamura, M. , Miyamoto, T. , Ohtori, S. , Matsumoto, Y. , Abe, T. , Takahashi, H. , Watanabe, K. , Hirano, T. , Ohashi, M. , … Yamazaki, M. (2021). Randomized trial of granulocyte colony‐stimulating factor for spinal cord injury. Brain, 144, 789–799. 10.1093/brain/awaa466 33764445 PMC8041047

[4] Lancaster, M. A. , & Knoblich, J. A. (2014). Organogenesis in a dish: Modeling development and disease using organoid technologies. Science, 345, 1247125. 10.1126/science.1247125 25035496

[5] Sato, T. , Vries, R. G. , Snippert, H. J. , Van De Wetering, M. , Barker, N. , Stange, D. E. , Van Es, J. H. , Abo, A. , Kujala, P. , Peters, P. J. , & Clevers, H. (2009). Single Lgr5 stem cells build crypt‐villus structures *in vitro* without a mesenchymal niche. Nature, 459, 262–265. 10.1038/nature07935 19329995

[6] Ootani, A. , Li, X. , Sangiorgi, E. , Ho, Q. T. , Ueno, H. , Toda, S. , Sugihara, H. , Fujimoto, K. , Weissman, I. L. , Capecchi, M. R. , & Kuo, C. J. (2009). Sustained in vitro intestinal epithelial culture within a Wnt‐dependent stem cell niche. Nature Medicine, 15, 701–706. 10.1038/nm.1951 PMC2919216 19398967

[7] Wilson, H. V. (1907). A new method by which sponges may be artificially reared. Science, 25, 912–915. 10.1126/science.25.649.912 17842577

[8] Barth, L. G. (1941). Neural differentiation without organizer. Journal of Experimental Zoology, 87, 371–383. 10.1002/jez.1400870303

[9] Holtfreter, J. (1944). Neural differentiation of ectoderm through exposure to saline solution. Journal of Experimental Zoology, 95, 307–343. 10.1002/jez.1400950303

[10] Hurtado, C. , & De Robertis, E. M. (2007). Neural induction in the absence of organizer in salamanders is mediated by MAPK. Developmental Biology, 307, 282–289. 10.1016/j.ydbio.2007.04.049 17540356 PMC2096472

[11] Moscona, A. (1952). Cell suspensions from organ rudiments of chick embryos. Experimental Cell Research, 3, 535–539. 10.1016/0014-4827(52)90077-3

[12] Moscona, A. , & Moscona, H. (1952). The dissociation and aggregation of cells from organ rudiments of the early chick embryo. Journal of Anatomy, 86, 287–301. 12980879 PMC1273752

[13] Weiss, P. , & Taylor, A. C. (1960). Reconstitution of complete organs from single‐cell suspensions of chick embryos in advanced stages of differentiation. Proceedings of the National Academy of Sciences of the United States of America, 46(1177), 1177–1185. 10.1073/pnas.46.9.1177 16590731 PMC223021

[14] Steinberg, M. S. (1964). The problem of adhesive selectivity in cellular interactions. In M. Locke (Ed.), Cellular membranes in development (pp. 321–366). Academic Press. 10.1016/B978-0-12-395533-3.50015-6

[15] Martin, G. R. (1981). Isolation of a pluripotent cell line from early mouse embryos cultured in medium conditioned by teratocarcinoma stem cells. Proceedings of the National Academy of Sciences of the United States of America, 78, 7634–7638. 10.1073/pnas.78.12.7634 6950406 PMC349323

[16] Thomson, J. A. , Itskovitz‐Eldor, J. , Shapiro, S. S. , Waknitz, M. A. , Swiergiel, J. J. , Marshall, V. S. , & Jones, J. M. (1998). Embryonic stem cell lines derived from human blastocysts. Science, 282, 1145–1147. 10.1126/science.282.5391.1145 9804556

[17] Evans, M. J. , & Kaufman, M. H. (1981). Establishment in culture of pluripotential cells from mouse embryos. Nature, 292, 154–156. 10.1038/292154a0 7242681

[18] Klug, M. G. , Soonpaa, M. H. , Koh, G. Y. , & Field, L. J. (1996). Genetically selected cardiomyocytes from differentiating embronic stem cells form stable intracardiac grafts. Journal of Clinical Investigation, 98, 216–224. 10.1172/JCI118769 8690796 PMC507419

[19] Lumelsky, N. , Blondel, O. , Laeng, P. , Velasco, I. , Ravin, R. , & Mckay, R. (2001). Differentiation of embryonic stem cells to insulin‐secreting structures similar to pancreatic islets. Science, 292, 1389–1394. 10.1126/science.1058866 11326082

[20] Bain, G. , Kitchens, D. , Yao, M. , Huettner, J. E. , & Gottlieb, D. I. (1995). Embryonic stem cells express neuronal properties *in vitro* . Developmental Biology, 168, 342–357. 10.1006/dbio.1995.1085 7729574

[21] Zhang, S.‐C. , Wernig, M. , Duncan, I. D. , Brüstle, O. , & Thomson, J. A. (2001). In vitro differentiation of transplantable neural precursors from human embryonic stem cells. Nature Biotechnology, 19, 1129–1133. 10.1038/nbt1201-1129 11731781

[22] Lee, S.‐H. , Lumelsky, N. , Studer, L. , Auerbach, J. M. , & Mckay, R. D. (2000). Efficient generation of midbrain and hindbrain neurons from mouse embryonic stem cells. Nature Biotechnology, 18, 675–679. 10.1038/76536 10835609

[23] Eisenstein, M. (2012). Stem cells: Don't believe the hype. Nature, 484, S5–S5. 10.1038/nature11107 22509506

[24] Luo, C. , Lancaster, M. A. , Castanon, R. , Nery, J. R. , Knoblich, J. A. , & Ecker, J. R. (2016). Cerebral organoids recapitulate epigenomic signatures of the human fetal brain. Cell Reports, 17, 3369–3384. 10.1016/j.celrep.2016.12.001 28009303 PMC5495578

[25] Stevens, L. C. (1960). Embryonic potency of embryoid bodies derived from a transplantable testicular teratoma of the mouse. Developmental Biology, 2, 285–297. 10.1016/0012-1606(60)90010-5 13834544

[26] Martin, G. R. , & Evans, M. J. (1975). Differentiation of clonal lines of teratocarcinoma cells: Formation of embryoid bodies in vitro. Proceedings of the National Academy of Sciences of the United States of America, 72, 1441–1445. 10.1073/pnas.72.4.1441 1055416 PMC432551

[27] Itskovitz‐Eldor, J. , Schuldiner, M. , Karsenti, D. , Eden, A. , Yanuka, O. , Amit, M. , Soreq, H. , & Benvenisty, N. (2000). Differentiation of human embryonic stem cells into embryoid bodies comprising the three embryonic germ layers. Molecular Medicine, 6, 88–95. 10.1007/BF03401776 10859025 PMC1949933

[28] Doetschman, T. C. , Eistetter, H. , Katz, M. , Schmidt, W. , & Kemler, R. (1985). The in vitro development of blastocyst‐derived embryonic stem cell lines: Formation of visceral yolk sac, blood islands and myocardium. Development (Cambridge, England), 87, 27–45. 10.1242/dev.87.1.27 3897439

[29] Mummery, C. L. , Zhang, J. , Ng, E. S. , Elliott, D. A. , Elefanty, A. G. , & Kamp, T. J. (2012). Differentiation of human ES and iPS cells to cardiomyocytes: A methods overview. Circulation Research, 111, 344–358. 10.1161/CIRCRESAHA.110.227512 22821908 PMC3578601

[30] Schuldiner, M. , Yanuka, O. , Itskovitz‐Eldor, J. , Melton, D. A. , & Benvenisty, N. (2000). Effects of eight growth factors on the differentiation of cells derived from human embryonic stem cells. Proceedings of the National Academy of Sciences of the United States of America, 97, 11307–11312. 10.1073/pnas.97.21.11307 11027332 PMC17196

[31] Watanabe, K. , Kamiya, D. , Nishiyama, A. , Katayama, T. , Nozaki, S. , Kawasaki, H. , Watanabe, Y. , Mizuseki, K. , & Sasai, Y. (2005). Directed differentiation of telencephalic precursors from embryonic stem cells. Nature Neuroscience, 8, 288–296. 10.1038/nn1402 15696161

[32] Ikeda, H. , Osakada, F. , Watanabe, K. , Mizuseki, K. , Haraguchi, T. , Miyoshi, H. , Kamiya, D. , Honda, Y. , Sasai, N. , Yoshimura, N. , Takahashi, M. , & Sasai, Y. (2005). Generation of Rx+/Pax6+ neural retinal precursors from embryonic stem cells. Proceedings of the National Academy of Sciences of the United States of America, 102, 11331–11336. 10.1073/pnas.0500010102 16076961 PMC1183536

[33] Su, H.‐L. , Muguruma, K. , Matsuo‐Takasaki, M. , Kengaku, M. , Watanabe, K. , & Sasai, Y. (2006). Generation of cerebellar neuron precursors from embryonic stem cells. Developmental Biology, 290, 287–296. 10.1016/j.ydbio.2005.11.010 16406324

[34] Eiraku, M. , Watanabe, K. , Matsuo‐Takasaki, M. , Kawada, M. , Yonemura, S. , Matsumura, M. , Wataya, T. , Nishiyama, A. , Muguruma, K. , & Sasai, Y. (2008). Self‐organized formation of polarized cortical tissues from ESCs and its active manipulation by extrinsic signals. Cell Stem Cell, 3, 519–532. 10.1016/j.stem.2008.09.002 18983967

[35] Spence, J. R. , Mayhew, C. N. , Rankin, S. A. , Kuhar, M. F. , Vallance, J. E. , Tolle, K. , Hoskins, E. E. , Kalinichenko, V. V. , Wells, S. I. , Zorn, A. M. , Shroyer, N. F. , & Wells, J. M. (2011). Directed differentiation of human pluripotent stem cells into intestinal tissue in vitro. Nature, 470, 105–109. 10.1038/nature09691 21151107 PMC3033971

[36] Eiraku, M. , Takata, N. , Ishibashi, H. , Kawada, M. , Sakakura, E. , Okuda, S. , Sekiguchi, K. , Adachi, T. , & Sasai, Y. (2011). Self‐organizing optic‐cup morphogenesis in three‐dimensional culture. Nature, 472, 51–56. 10.1038/nature09941 21475194

[37] Koehler, K. R. , Mikosz, A. M. , Molosh, A. I. , Patel, D. , & Hashino, E. (2013). Generation of inner ear sensory epithelia from pluripotent stem cells in 3D culture. Nature, 500, 217–221. 10.1038/nature12298 23842490 PMC3739998

[38] Sasai, Y. (2013). Cytosystems dynamics in self‐organization of tissue architecture. Nature, 493, 318–326. 10.1038/nature11859 23325214

[39] Nakano, T. , Ando, S. , Takata, N. , Kawada, M. , Muguruma, K. , Sekiguchi, K. , Saito, K. , Yonemura, S. , Eiraku, M. , & Sasai, Y. (2012). Self‐formation of optic cups and storable stratified neural retina from human ESCs. Cell Stem Cell, 10, 771–785. 10.1016/j.stem.2012.05.009 22704518

[40] Muñoz‐Sanjuán, I. , & Brivanlou, A. H. (2002). Neural induction, the default model and embryonic stem cells. Nature Reviews Neuroscience, 3, 271–280. 10.1038/nrn786 11967557

[41] Schulz, T. C. , Palmarini, G. M. , Noggle, S. A. , Weiler, D. A. , Mitalipova, M. M. , & Condie, B. G. (2003). Directed neuronal differentiation of human embryonic stem cells. BMC Neuroscience, 4, 27. 10.1186/1471-2202-4-27 14572319 PMC272931

[42] Lancaster, M. A. , Renner, M. , Martin, C.‐A. , Wenzel, D. , Bicknell, L. S. , Hurles, M. E. , Homfray, T. , Penninger, J. M. , Jackson, A. P. , & Knoblich, J. A. (2013). Cerebral organoids model human brain development and microcephaly. Nature, 501, 373–379. 10.1038/nature12517 23995685 PMC3817409

[43] Pașca, S. P. , Arlotta, P. , Bateup, H. S. , Camp, J. G. , Cappello, S. , Gage, F. H. , Knoblich, J. A. , Kriegstein, A. R. , Lancaster, M. A. , Ming, G.‐L. , Muotri, A. R. , Park, I.‐H. , Reiner, O. , Song, H. , Studer, L. , Temple, S. , Testa, G. , Treutlein, B. , & Vaccarino, F. M. (2022). A nomenclature consensus for nervous system organoids and assembloids. Nature, 609, 907–910. 10.1038/s41586-022-05219-6 36171373 PMC10571504

[44] Renner, M. , Lancaster, M. A. , Bian, S. , Choi, H. , Ku, T. , Peer, A. , Chung, K. , & Knoblich, J. A. (2017). Self‐organized developmental patterning and differentiation in cerebral organoids. The EMBO Journal, 36, 1316–1329. 10.15252/embj.201694700 28283582 PMC5430225

[45] Kim, H. , Im, I. , Jeon, J. S. , Kang, E.‐H. , Lee, H.‐A. , Jo, S. , Kim, J.‐W. , Woo, D.‐H. , Choi, Y. J. , Kim, H. J. , Han, J.‐S. , Lee, B.‐S. , Kim, J.‐H. , Kim, S. K. , & Park, H.‐J. (2022). Development of human pluripotent stem cell‐derived hepatic organoids as an alternative model for drug safety assessment. Biomaterials, 286, 121575. 10.1016/j.biomaterials.2022.121575 35598335

[46] Kim, J. , Koo, B.‐K. , & Knoblich, J. A. (2020). Human organoids: Model systems for human biology and medicine. Nature Reviews Molecular Cell Biology, 21, 571–584. 10.1038/s41580-020-0259-3 32636524 PMC7339799

[47] Antonica, F. , Kasprzyk, D. F. , Opitz, R. , Iacovino, M. , Liao, X.‐H. , Dumitrescu, A. M. , Refetoff, S. , Peremans, K. , Manto, M. , Kyba, M. , & Costagliola, S. (2012). Generation of functional thyroid from embryonic stem cells. Nature, 491, 66–71. 10.1038/nature11525 23051751 PMC3687105

[48] Mccracken, K. W. , Catá, E. M. , Crawford, C. M. , Sinagoga, K. L. , Schumacher, M. , Rockich, B. E. , Tsai, Y.‐H. , Mayhew, C. N. , Spence, J. R. , Zavros, Y. , & Wells, J. M. (2014). Modelling human development and disease in pluripotent stem‐cell‐derived gastric organoids. Nature, 516, 400–404. 10.1038/nature13863 25363776 PMC4270898

[49] Dye, B. R. , Hill, D. R. , Ferguson, M. A. , Tsai, Y.‐H. , Nagy, M. S. , Dyal, R. , Wells, J. M. , Mayhew, C. N. , Nattiv, R. , Klein, O. D. , White, E. S. , Deutsch, G. H. , & Spence, J. R. (2015). In vitro generation of human pluripotent stem cell derived lung organoids. eLife, 4, e05098. 10.7554/eLife.05098 25803487 PMC4370217

[50] Lee, J. , Rabbani, C. C. , Gao, H. , Steinhart, M. R. , Woodruff, B. M. , Pflum, Z. E. , Kim, A. , Heller, S. , Liu, Y. , Shipchandler, T. Z. , & Koehler, K. R. (2020). Hair‐bearing human skin generated entirely from pluripotent stem cells. Nature, 582, 399–404. 10.1038/s41586-020-2352-3 32494013 PMC7593871

[51] Hofbauer, P. , Jahnel, S. M. , Papai, N. , Giesshammer, M. , Deyett, A. , Schmidt, C. , Penc, M. , Tavernini, K. , Grdseloff, N. , Meledeth, C. , Ginistrelli, L. C. , Ctortecka, C. , Šalic, Š. , Novatchkova, M. , & Mendjan, S. (2021). Cardioids reveal self‐organizing principles of human cardiogenesis. Cell, 184, 3299–3317.e22. 10.1016/j.cell.2021.04.034 34019794

[52] Karvas, R. M. , Khan, S. A. , Verma, S. , Yin, Y. , Kulkarni, D. , Dong, C. , Park, K.‐M. , Chew, B. , Sane, E. , Fischer, L. A. , Kumar, D. , Ma, L. , Boon, A. C. M. , Dietmann, S. , Mysorekar, I. U. , & Theunissen, T. W. (2022). Stem‐cell‐derived trophoblast organoids model human placental development and susceptibility to emerging pathogens. Cell Stem Cell, 29, 810–825.e8. 10.1016/j.stem.2022.04.004 35523141 PMC9136997

[53] Wimmer, R. A. , Leopoldi, A. , Aichinger, M. , Wick, N. , Hantusch, B. , Novatchkova, M. , Taubenschmid, J. , Hämmerle, M. , Esk, C. , Bagley, J. A. , Lindenhofer, D. , Chen, G. , Boehm, M. , Agu, C. A. , Yang, F. , Fu, B. , Zuber, J. , Knoblich, J. A. , Kerjaschki, D. , & Penninger, J. M. (2019). Human blood vessel organoids as a model of diabetic vasculopathy. Nature, 565, 505–510. 10.1038/s41586-018-0858-8 30651639 PMC7116578

[54] Xia, Y. , Nivet, E. , Sancho‐Martinez, I. , Gallegos, T. , Suzuki, K. , Okamura, D. , Wu, M.‐Z. , Dubova, I. , Esteban, C. R. , Montserrat, N. , Campistol, J. M. , & Belmonte, J. C. I. (2013). Directed differentiation of human pluripotent cells to ureteric bud kidney progenitor‐like cells. Nature Cell Biology, 15, 1507–1515. 10.1038/ncb2872 24240476

[55] Taguchi, A. , Kaku, Y. , Ohmori, T. , Sharmin, S. , Ogawa, M. , Sasaki, H. , & Nishinakamura, R. (2014). Redefining the in vivo origin of metanephric nephron progenitors enables generation of complex kidney structures from pluripotent stem cells. Cell Stem Cell, 14, 53–67. 10.1016/j.stem.2013.11.010 24332837

[56] Takasato, M. , Er, P. X. , Becroft, M. , Vanslambrouck, J. M. , Stanley, E. G. , Elefanty, A. G. , & Little, M. H. (2014). Directing human embryonic stem cell differentiation towards a renal lineage generates a self‐organizing kidney. Nature Cell Biology, 16, 118–126. 10.1038/ncb2894 24335651

[57] Takasato, M. , Er, P. X. , Chiu, H. S. , Maier, B. , Baillie, G. J. , Ferguson, C. , Parton, R. G. , Wolvetang, E. J. , Roost, M. S. , Chuva De Sousa Lopes, S. M. , & Little, M. H. (2015). Kidney organoids from human iPS cells contain multiple lineages and model human nephrogenesis. Nature, 526, 564–568. 10.1038/nature15695 26444236

[58] Van Den Brink, S. C. , Baillie‐Johnson, P. , Balayo, T. , Hadjantonakis, A.‐K. , Nowotschin, S. , Turner, D. A. , & Martinez Arias, A. (2014). Symmetry breaking, germ layer specification and axial organisation in aggregates of mouse embryonic stem cells. Development (Cambridge, England), 41, 4231–4242. 10.1242/dev.113001 PMC4302915 25371360

[59] Moris, N. , Anlas, K. , Van Den Brink, S. C. , Alemany, A. , Schröder, J. , Ghimire, S. , Balayo, T. , Van Oudenaarden, A. , & Martinez Arias, A. (2020). An in vitro model of early anteroposterior organization during human development. Nature, 582, 410–415. 10.1038/s41586-020-2383-9 32528178

[60] Veenvliet, J. V. , Bolondi, A. , Kretzmer, H. , Haut, L. , Scholze‐Wittler, M. , Schifferl, D. , Koch, F. , Guignard, L. , Kumar, A. S. , Pustet, M. , Heimann, S. , Buschow, R. , Wittler, L. , Timmermann, B. , Meissner, A. , & Herrmann, B. G. (2020). Mouse embryonic stem cells self‐organize into trunk‐like structures with neural tube and somites. Science, 370, eaba4937. 10.1126/science.aba4937 33303587

[61] Harrison, S. E. , Sozen, B. , Christodoulou, N. , Kyprianou, C. , & Zernicka‐Goetz, M. (2017). Assembly of embryonic and extraembryonic stem cells to mimic embryogenesis in vitro. Science, 356, eaal1810. 10.1126/science.aal1810 28254784

[62] Tarazi, S. , Aguilera‐Castrejon, A. , Joubran, C. , Ghanem, N. , Ashouokhi, S. , Roncato, F. , Wildschutz, E. , Haddad, M. , Oldak, B. , Gomez‐Cesar, E. , Livnat, N. , Viukov, S. , Lokshtanov, D. , Naveh‐Tassa, S. , Rose, M. , Hanna, S. , Raanan, C. , Brenner, O. , Kedmi, M. , … Hanna, J. H. (2022). Post‐gastrulation synthetic embryos generated *ex utero* from mouse naive ESCs. Cell, 185, 3290–3306.e25. 10.1016/j.cell.2022.07.028 35988542 PMC9439721

[63] Oldak, B. , Wildschutz, E. , Bondarenko, V. , Comar, M.‐Y. , Zhao, C. , Aguilera‐Castrejon, A. , Tarazi, S. , Viukov, S. , Pham, T. X. A. , Ashouokhi, S. , Lokshtanov, D. , Roncato, F. , Ariel, E. , Rose, M. , Livnat, N. , Shani, T. , Joubran, C. , Cohen, R. , Addadi, Y. , … Hanna, J. H. (2023). Complete human day 14 post‐implantation embryo models from naive ES cells. Nature, 622, 562–573. 10.1038/s41586-023-06604-5 37673118 PMC10584686

[64] Weatherbee, B. A. T. , Gantner, C. W. , Iwamoto‐Stohl, L. K. , Daza, R. M. , Hamazaki, N. , Shendure, J. , & Zernicka‐Goetz, M. (2023). Pluripotent stem cell‐derived model of the post‐implantation human embryo. Nature, 622, 584–593. 10.1038/s41586-023-06368-y 37369347 PMC10584688

[65] Amadei, G. , Handford, C. E. , Qiu, C. , De Jonghe, J. , Greenfeld, H. , Tran, M. , Martin, B. K. , Chen, D.‐Y. , Aguilera‐Castrejon, A. , Hanna, J. H. , Elowitz, M. B. , Hollfelder, F. , Shendure, J. , Glover, D. M. , & Zernicka‐Goetz, M. (2022). Embryo model completes gastrulation to neurulation and organogenesis. Nature, 610, 143–153. 10.1038/s41586-022-05246-3 36007540 PMC9534772

[66] Sozen, B. , Amadei, G. , Cox, A. , Wang, R. , Na, E. , Czukiewska, S. , Chappell, L. , Voet, T. , Michel, G. , Jing, N. , Glover, D. M. , & Zernicka‐Goetz, M. (2018). Self‐assembly of embryonic and two extra‐embryonic stem cell types into gastrulating embryo‐like structures. Nature Cell Biology, 20, 979–989. 10.1038/s41556-018-0147-7 30038254

[67] Alvarez‐Buylla, A. (2014). Yoshiki Sasai (1962–2014). Nature, 513, 34–34. 10.1038/513034a 25186892

[68] Little, M. H. , & Combes, A. N. (2019). Kidney organoids: Accurate models or fortunate accidents. Genes & Development, 33, 1319–1345. 10.1101/gad.329573.119 31575677 PMC6771389

[69] Lancaster, M. A. (2021). Brain organoids: A new frontier of human neuroscience research. Seminars in Cell & Developmental Biology, 111, 1–3. 10.1016/j.semcdb.2020.10.011 33158731

[70] Workman, M. J. , Mahe, M. M. , Trisno, S. , Poling, H. M. , Watson, C. L. , Sundaram, N. , Chang, C.‐F. , Schiesser, J. , Aubert, P. , Stanley, E. G. , Elefanty, A. G. , Miyaoka, Y. , Mandegar, M. A. , Conklin, B. R. , Neunlist, M. , Brugmann, S. A. , Helmrath, M. A. , & Wells, J. M. (2017). Engineered human pluripotent‐stem‐cell‐derived intestinal tissues with a functional enteric nervous system. Nature Medicine, 23, 49–59. 10.1038/nm.4233 PMC5562951 27869805

[71] Tran, T. , Song, C. J. , Nguyen, T. , Cheng, S.‐Y. , Mcmahon, J. A. , Yang, R. , Guo, Q. , Der, B. , Lindström, N. O. , Lin, D. C.‐H. , & Mcmahon, A. P. (2022). A scalable organoid model of human autosomal dominant polycystic kidney disease for disease mechanism and drug discovery. Cell Stem Cell, 29, 1083–1101.e7. 10.1016/j.stem.2022.06.005 35803227 PMC11088748

[72] Mariani, J. , Coppola, G. , Zhang, P. , Abyzov, A. , Provini, L. , Tomasini, L. , Amenduni, M. , Szekely, A. , Palejev, D. , Wilson, M. , Gerstein, M. , Grigorenko, E. L. , Chawarska, K. , Pelphrey, K. A. , Howe, J. R. , & Vaccarino, F. M. (2015). FOXG1‐dependent dysregulation of GABA/glutamate neuron differentiation in autism spectrum disorders. Cell, 162, 375–390. 10.1016/j.cell.2015.06.034 26186191 PMC4519016

[73] Steinberg, D. J. , Repudi, S. , Saleem, A. , Kustanovich, I. , Viukov, S. , Abudiab, B. , Banne, E. , Mahajnah, M. , Hanna, J. H. , Stern, S. , Carlen, P. L. , & Aqeilan, R. I. (2021). Modeling genetic epileptic encephalopathies using brain organoids. EMBO Molecular Medicine, 13, e13610. 10.15252/emmm.202013610 34268881 PMC8350905

[74] Eichmüller, O. L. , Corsini, N. S. , Vértesy, Á. , Morassut, I. , Scholl, T. , Gruber, V.‐E. , Peer, A. M. , Chu, J. , Novatchkova, M. , Hainfellner, J. A. , Paredes, M. F. , Feucht, M. , & Knoblich, J. A. (2022). Amplification of human interneuron progenitors promotes brain tumors and neurological defects. Science, 375, eabf5546. 10.1126/science.abf5546 35084981 PMC7613689

[75] Birey, F. , Li, M.‐Y. , Gordon, A. , Thete, M. V. , Valencia, A. M. , Revah, O. , Paşca, A. M. , Geschwind, D. H. , & Paşca, S. P. (2022). Dissecting the molecular basis of human interneuron migration in forebrain assembloids from Timothy syndrome. Cell Stem Cell, 29, 248–264.e7. 10.1016/j.stem.2021.11.011 34990580 PMC13492730

[76] Chen, X. , Birey, F. , Li, M.‐Y. , Revah, O. , Levy, R. , Thete, M. V. , Reis, N. , Kaganovsky, K. , Onesto, M. , Sakai, N. , Hudacova, Z. , Hao, J. , Meng, X. , Nishino, S. , Huguenard, J. , & Pașca, S. P. (2024). Antisense oligonucleotide therapeutic approach for Timothy syndrome. Nature, 628, 818–825. 10.1038/s41586-024-07310-6 38658687 PMC11043036

[77] Qian, X. , Nguyen, H. N. , Song, M. M. , Hadiono, C. , Ogden, S. C. , Hammack, C. , Yao, B. , Hamersky, G. R. , Jacob, F. , Zhong, C. , Yoon, K.‐J. , Jeang, W. , Lin, L. , Li, Y. , Thakor, J. , Berg, D. A. , Zhang, C. , Kang, E. , Chickering, M. , … Ming, G. L. (2016). Brain‐region‐specific organoids using mini‐bioreactors for modeling ZIKV exposure. Cell, 165, 1238–1254. 10.1016/j.cell.2016.04.032 27118425 PMC4900885

[78] Kumar, S. V. , Er, P. X. , Lawlor, K. T. , Motazedian, A. , Scurr, M. , Ghobrial, I. , Combes, A. N. , Zappia, L. , Oshlack, A. , Stanley, E. G. , & Little, M. H. (2019). Kidney micro‐organoids in suspension culture as a scalable source of human pluripotent stem cell‐derived kidney cells. Development (Cambridge, England), 146, dev172361. 10.1242/dev.172361 30846463 PMC6432662

[79] Yoon, S.‐J. , Elahi, L. S. , Pașca, A. M. , Marton, R. M. , Gordon, A. , Revah, O. , Miura, Y. , Walczak, E. M. , Holdgate, G. M. , Fan, H. C. , Huguenard, J. R. , Geschwind, D. H. , & Pașca, S. P. (2019). Reliability of human cortical organoid generation. Nature Methods, 16, 75–78. 10.1038/s41592-018-0255-0 30573846 PMC6677388

[80] Chiaradia, I. , Imaz‐Rosshandler, I. , Nilges, B. S. , Boulanger, J. , Pellegrini, L. , Das, R. , Kashikar, N. D. , & Lancaster, M. A. (2023). Tissue morphology influences the temporal program of human brain organoid development. Cell Stem Cell, 30, 1351–1367.e10. 10.1016/j.stem.2023.09.003 37802039 PMC10765088

[81] Birey, F. , Andersen, J. , Makinson, C. D. , Islam, S. , Wei, W. , Huber, N. , Fan, H. C. , Metzler, K. R. C. , Panagiotakos, G. , Thom, N. , O'rourke, N. A. , Steinmetz, L. M. , Bernstein, J. A. , Hallmayer, J. , Huguenard, J. R. , & Paşca, S. P. (2017). Assembly of functionally integrated human forebrain spheroids. Nature, 545, 54–59. 10.1038/nature22330 28445465 PMC5805137

[82] Koike, H. , Iwasawa, K. , Ouchi, R. , Maezawa, M. , Giesbrecht, K. , Saiki, N. , Ferguson, A. , Kimura, M. , Thompson, W. L. , Wells, J. M. , Zorn, A. M. , & Takebe, T. (2019). Modelling human hepato‐biliary‐pancreatic organogenesis from the foregut–midgut boundary. Nature, 574, 112–116. 10.1038/s41586-019-1598-0 31554966 PMC7643931

[83] Faustino Martins, J.‐M. , Fischer, C. , Urzi, A. , Vidal, R. , Kunz, S. , Ruffault, P.‐L. , Kabuss, L. , Hube, I. , Gazzerro, E. , Birchmeier, C. , Spuler, S. , Sauer, S. , & Gouti, M. (2020). Self‐organizing 3D human trunk neuromuscular organoids. Cell Stem Cell, 26, 172–186.e6. 10.1016/j.stem.2019.12.007 31956040

[84] Cuevas, E. , Holder, D. L. , Alshehri, A. H. , Tréguier, J. , Lakowski, J. , & Sowden, J. C. (2021). NRL−/− gene edited human embryonic stem cells generate rod‐deficient retinal organoids enriched in S‐cone‐like photoreceptors. Stem Cells, 39, 414–428. 10.1002/stem.3325 33400844 PMC8438615

[85] Benito‐Kwiecinski, S. , Giandomenico, S. L. , Sutcliffe, M. , Riis, E. S. , Freire‐Pritchett, P. , Kelava, I. , Wunderlich, S. , Martin, U. , Wray, G. A. , Mcdole, K. , & Lancaster, M. A. (2021). An early cell shape transition drives evolutionary expansion of the human forebrain. Cell, 184, 2084–2102. e19. 10.1016/j.cell.2021.02.050 33765444 PMC8054913

[86] Blair, J. D. , Hockemeyer, D. , & Bateup, H. S. (2018). Genetically engineered human cortical spheroid models of tuberous sclerosis. Nature Medicine, 24, 1568–1578. 10.1038/s41591-018-0139-y PMC6261470 30127391

[87] Homan, K. A. , Gupta, N. , Kroll, K. T. , Kolesky, D. B. , Skylar‐Scott, M. , Miyoshi, T. , Mau, D. , Valerius, M. T. , Ferrante, T. , Bonventre, J. V. , Lewis, J. A. , & Morizane, R. (2019). Flow‐enhanced vascularization and maturation of kidney organoids in vitro. Nature Methods, 16, 255–262. 10.1038/s41592-019-0325-y 30742039 PMC6488032

[88] Xue, X. , Kim, Y. S. , Ponce‐Arias, A.‐I. , O'laughlin, R. , Yan, R. Z. , Kobayashi, N. , Tshuva, R. Y. , Tsai, Y.‐H. , Sun, S. , Zheng, Y. , Liu, Y. , Wong, F. C. K. , Surani, A. , Spence, J. R. , Song, H. , Ming, G.‐L. , Reiner, O. , & Fu, J. (2024). A patterned human neural tube model using microfluidic gradients. Nature, 628, 391–399. 10.1038/s41586-024-07204-7 38408487 PMC11006583

[89] Moerkens, R. , Mooiweer, J. , Ramírez‐Sánchez, A. D. , Oelen, R. , Franke, L. , Wijmenga, C. , Barrett, R. J. , Jonkers, I. H. , & Withoff, S. (2024). An iPSC‐derived small intestine‐on‐chip with self‐organizing epithelial, mesenchymal, and neural cells. Cell Reports, 43, 114247. 10.1016/j.celrep.2024.114247 38907996

[90] Park, S. E. , Georgescu, A. , & Huh, D. (2019). Organoids‐on‐a‐chip. Science, 364, 960–965. 10.1126/science.aaw7894 31171693 PMC7764943

[91] Gjorevski, N. , Nikolaev, M. , Brown, T. E. , Mitrofanova, O. , Brandenberg, N. , Delrio, F. W. , Yavitt, F. M. , Liberali, P. , Anseth, K. S. , & Lutolf, M. P. (2022). Tissue geometry drives deterministic organoid patterning. Science, 375, eaaw9021. 10.1126/science.aaw9021 34990240 PMC9131435

[92] Lancaster, M. A. , Corsini, N. S. , Wolfinger, S. , Gustafson, E. H. , Phillips, A. W. , Burkard, T. R. , Otani, T. , Livesey, F. J. , & Knoblich, J. A. (2017). Guided self‐organization and cortical plate formation in human brain organoids. Nature Biotechnology, 35, 659–666. 10.1038/nbt.3906 PMC5824977 28562594

[93] Giandomenico, S. L. , Mierau, S. B. , Gibbons, G. M. , Wenger, L. M. D. , Masullo, L. , Sit, T. , Sutcliffe, M. , Boulanger, J. , Tripodi, M. , Derivery, E. , Paulsen, O. , Lakatos, A. , & Lancaster, M. A. (2019). Cerebral organoids at the air–liquid interface generate diverse nerve tracts with functional output. Nature Neuroscience, 22, 669–679. 10.1038/s41593-019-0350-2 30886407 PMC6436729

[94] Altman, J. , & Bayer, S. A. , (2015) Development of the human neocortex. A review and interpretation of the histological record. Laboratory of Developmental Neurobiology, Inc. http://neurondevelopment.org/the‐development‐of‐the‐neocortex/

[95] Paşca, A. M. , Sloan, S. A. , Clarke, L. E. , Tian, Y. , Makinson, C. D. , Huber, N. , Kim, C. H. , Park, J.‐Y. , O'rourke, N. A. , Nguyen, K. D. , Smith, S. J. , Huguenard, J. R. , Geschwind, D. H. , Barres, B. A. , & Paşca, S. P. (2015). Functional cortical neurons and astrocytes from human pluripotent stem cells in 3D culture. Nature Methods, 12, 671–678. 10.1038/nmeth.3415 26005811 PMC4489980

[96] Pellegrini, L. , Bonfio, C. , Chadwick, J. , Begum, F. , Skehel, M. , & Lancaster, M. A. (2020). Human CNS barrier‐forming organoids with cerebrospinal fluid production. Science, 369, eaaz5626. 10.1126/science.aaz5626 32527923 PMC7116154

[97] Sakaguchi, H. , Kadoshima, T. , Soen, M. , Narii, N. , Ishida, Y. , Ohgushi, M. , Takahashi, J. , Eiraku, M. , & Sasai, Y. (2015). Generation of functional hippocampal neurons from self‐organizing human embryonic stem cell‐derived dorsomedial telencephalic tissue. Nature Communications, 6, 8896. 10.1038/ncomms9896 PMC4660208 26573335

[98] Bagley, J. A. , Reumann, D. , Bian, S. , Lévi‐Strauss, J. , & Knoblich, J. A. (2017). Fused cerebral organoids model interactions between brain regions. Nature Methods, 14, 743–751. 10.1038/nmeth.4304 28504681 PMC5540177

